# Low use of placebo in comparative drug rcts in migraine. Are clinical investigators unaware of basic methodological issues?

**DOI:** 10.1186/1129-2377-14-S1-P135

**Published:** 2013-02-21

**Authors:** PC Tfelt-Hansen, A Hougaard

**Affiliations:** 1 Danish Headache Centre, Denmark

## 

If feasible placebo should be included in randomized, controlled trials (RCTs) comparing two presumable active drugs. Inclusion of placebo in comparative RCTs was recommended generally by FDA in 1982, by the International Headache Society for migraine in 1991, and by the European Medicines Agency (EMEA) for migraine in 2007.

## Methods

We searched the world migraine literature from 2002 to 2011 for comparative drug RCTs of acute and prophylactic treatment of migraine. We primarily registered whether placebo was used or not.

## Results

In 67 acute comparative RCTs placebo was included in 26 RCTs and in 26 prophylactic comparative RCTs placebo was included in only 2 RCTs [sic]. For the acute RCTs without placebo (n=41) the median number of patients was 76 (range: 12-2,436) and for acute RCTs with placebo (505) the median number of patients was 505 (range 42 -2,113).

## Discussion

Recommendations by FDA, IHS and EMEA for inclusion of placebo in comparative RCTs are plenty but, seemingly, clinical investigators and the pharmaceutical industry need to be informed more about why these rules should be followed. The most important fact to be aware of is the “effectiveness” of placebo in drug RCTs in migraine. Thus, in acute RCTs with oral triptans the placebo response for headache relief at 2 h varied from 17 to 50% [1]. In prophylactic RCTs the placebo-response is usually in the 20 to 40% range but 50% and even 70% [2]have been reported. So if two “active” drugs both show similar response rates of 45% and 49% in a prophylactic RCT in migraine one cannot speak of equivalence even with narrow confidence intervals because no “pharmacological “ effect has been demonstrated in the patient sample. What is observed in this RCT could be pure placebo effect or time-effect (regression toward the mean). Thus the RCT has been performed in vain. In addition, placebo control is needed for characterization of the tolerability of new drugs. Finally, a comparative “equivalence” RCT without placebo cannot be used in meta-analysis and does not add to sound scientific knowledge of the drug.
